# The effect of acute aerobic exercise on inhibitory control in young adults with trait anxiety: an ERP study

**DOI:** 10.3389/fpubh.2026.1944238

**Published:** 2026-09-09

**Authors:** Li Yao, Shuliang Huang, Jianqi Bai, Zhicheng Zhu

**Affiliations:** 1 School of Sports Training, Chengdu Sport University, Chengdu, China; 2 Institute of Physical Education, Xinyu University, Xinyu, China

**Keywords:** aerobic exercise, event-related potentials, inhibitory control, Stroop task, trait anxiety

## Abstract

**Background:**

Regular physical activity is known to benefit emotion and cognition, and acute moderate-intensity aerobic exercise has been shown to improve executive function, particularly inhibitory control, in healthy populations. However, whether these effects generalize across individuals with different levels of trait anxiety remains unclear. The present study investigated the effects of acute moderate-intensity aerobic exercise on state anxiety and inhibitory control in young adults with low and high trait anxiety using the Stroop task, with a particular focus on the temporal neural processes associated with Stroop performance.

**Methods:**

Based on trait anxiety scores, participants were assigned to either the low trait anxiety (LTA, *n* = 21) or high trait anxiety (HTA, *n* = 21) group. All participants completed both a 30-min moderate-intensity aerobic exercise session and a resting control session. Following each session, participants completed a state-anxiety assessment and performed a color-word Stroop task while stimulus-locked event-related potentials (ERPs) were recorded.

**Results:**

Acute moderate-intensity aerobic exercise reduced state anxiety in the HTA group but not in the LTA group. Exercise also shortened response times and reduced error rates across both congruent and incongruent Stroop conditions in both groups. At the neural level, acute aerobic exercise was associated with less negative N400 amplitudes and larger sustained positive potential (SP) amplitudes during the Stroop task in both groups.

**Conclusion:**

A single bout of moderate-intensity aerobic exercise selectively reduced state anxiety in individuals with HTA and broadly improved Stroop performance across both trait anxiety groups. These behavioral changes were accompanied by exercise-related modulation of N400 and SP amplitudes, suggesting alterations in neural processes associated with conflict-related cognitive control.

## 1 Introduction

Physical exercise induces functional adaptations across multiple biological systems and contributes to mental and brain health. Previous studies have examined the effects of exercise type, frequency, duration, and intensity on mood and cognition (1, 2). Evidence from acute exercise studies suggests that even a single bout lasting more than 10 min can produce transient benefits across multiple domains of cognition and brain function (3–6). In particular, 20–40 min of moderate-intensity aerobic exercise has been consistently associated with improved cognitive performance and executive function, including inhibitory control in healthy individuals across the lifespan (7, 8) and in populations with neurodevelopmental disorders such as attention deficit hyperactivity disorder (5, 6).

Anxiety is an emotional response characterized by tension, worry, and heightened physiological arousal under conditions of uncertainty, danger, or potential threat. It can be conceptualized as both a transient psychophysiological state (state anxiety) and a relatively stable personality disposition (trait anxiety) (9). State anxiety reflects short-term psychological and physiological reactions, whereas trait anxiety reflects individual differences in the tendency to experience anxiety across situations. Recent perspectives suggest that anxiety-related cognitive alterations should be understood within an integrated affective–cognitive framework, in which emotional processes and executive control interact dynamically (10). Anxiety is characterized not only by altered emotional responses but also by inefficient allocation of cognitive resources, whereby worry and threat-related processing compete with goal-directed executive control (9, 11, 12). Importantly, these anxiety-related disruptions in attentional and cognitive control are not necessarily fixed deficits and may therefore represent modifiable targets for interventions aimed at strengthening executive control processes.

Acute aerobic exercise represents one such intervention, as it simultaneously affects affective regulation and executive functioning. By modifying physiological arousal and cognitive resource allocation, exercise may reduce anxiety-related interference while supporting goal-directed cognitive control (10, 13). Importantly, previous studies have shown that acute aerobic exercise can enhance inhibitory control in both high- and low-anxious individuals (14), suggesting that exercise-related cognitive benefits may occur broadly across anxiety levels. Furthermore, individuals with high trait anxiety have been found to exhibit greater sensitivity to conflict-related interference (15), yet this vulnerability does not necessarily imply reduced responsiveness to exercise-induced cognitive benefits (11). Thus, acute exercise may benefit inhibitory control across trait anxiety levels, although the magnitude of these effects may vary as a function of individual differences in anxiety.

Event-related potentials (ERPs), because of their high temporal resolution, are widely used to examine cognitive processes in individuals with trait anxiety (15, 16) and to characterize neural responses to acute exercise beyond overt behavioral performance (7, 17). Previous ERP studies has shown that trait anxiety is associated with altered conflict-related N400 responses during Stroop performance (15). In the Stroop paradigm, the N400 has been associated with conflict-monitoring processing during stimulus evaluation and response selection (15). A study employing the Stroop task found that improved task performance following acute moderate-intensity aerobic exercise was accompanied by less negative N400 amplitudes (7). Thus, acute aerobic exercise may influence neural processes involved in conflict-related cognitive control (7). A later sustained positive potential (SP) following the N400 has been proposed to reflect later stages of conflict detection and resolution (15). In the Stroop task, incongruent trials involving stimulus–response conflicts, engages greater cognitive demands to inhibit irrelevant information and elicits larger SP amplitude than the congruent trials (15, 18). Therefore, SP amplitude may be sensitive to exercise-related modulation of later-stage conflict processing.

However, several important questions remain unresolved. First, although acute aerobic exercise has been shown to reduce state anxiety, with larger affective benefits often observed in individuals with higher baseline anxiety levels (19), it remains unclear whether acute exercise can effectively alleviate state anxiety in individuals with elevated trait anxiety. Second, previous ERP studies have demonstrated that trait anxiety is associated with altered conflict-related neural responses, particularly in components such as the N400 and SP that are related to conflict monitoring and later-stage cognitive control processes (15, 18). In addition, acute aerobic exercise has been shown to improve Stroop performance and modulate ERP indices associated with cognitive control, including reduced conflict-related N400 amplitudes (7). However, whether acute exercise modulates these conflict-related neural processes differently across individuals with different levels of trait anxiety remains unclear.

The present study examined the acute effects of a 30 min bout of moderate-intensity aerobic exercise on state anxiety, inhibitory control assessed by the Stroop task, as well as the associated temporal dynamics in individuals with different levels of trait anxiety. Participants with high trait anxiety (HTA) and low trait anxiety (LTA) completed a 30 min moderate-intensity aerobic exercise session on a cycle ergometer and a resting control session on separate days. State anxiety was assessed and Stroop performance was measured following each session, while stimulus-locked ERPs were recorded during the Stroop task. Based on previous evidence showing that acute exercise may reduce state anxiety, particularly among individuals with elevated anxiety levels (19), we hypothesized that acute moderate-intensity aerobic exercise would reduce state anxiety, with potentially stronger effects in the HTA group. Based on previous evidence showing broad cognitive benefits of acute aerobic exercise across populations, including individuals with different anxiety levels (7, 14), we hypothesized that acute moderate-intensity aerobic exercise would improve inhibitory control performance in both LTA and HTA groups. Furthermore, we hypothesized that behavioral improvements following acute exercise would be accompanied by less negative N400 amplitudes and enhanced SP amplitudes, reflecting exercise-related modulation of conflict-related processing.

## 2 Methods

### 2.1 Participants

A total of 871 undergraduate students from Chengdu Sport University completed the Chinese version of trait subscale of the Spielberger’s State–Trait Anxiety Inventory (STAI-T) (20, 21). Based on previous research, individuals within the highest and lowest 27% of the STAI-T score distribution were classified as the HTA and LTA groups, respectively (22). Therefore, participants with STAI-T scores ≥48 were assigned to the HTA group, whereas participants with scores ≤38 were assigned to the LTA group. The sample size was estimated *a priori* using G*Power 3.1. A medium effect size (*f* = 0.25) was assumed based on previous acute-exercise studies reporting beneficial effects on inhibitory-control performance and in accordance with a comparable ERP study of acute moderate-intensity exercise (11, 23). Following the power-analysis parameters used by Aly and Kojima (23), a repeated-measures correlation of 0.75 was assumed. With a power of 0.95, an alpha level of 0.05, two groups, and two repeated measurements, the required sample size was 30 participants. Inclusion criteria were as follows: voluntary participation, right-handledness, age between 18 and 25 years, a normal or corrected-to-normal vision, normal color vision, and no self-reported history of mental disorders and physical disability. 42 healthy students (14 females and 28 males) were recruited to participate in the experiment and assigned to the LTA (*n* = 21; mean age = 19.90 ± 0.25) or HTA (*n* = 21; mean age = 20.29 ± 0.34) group according to their STAI-T scores.

### 2.2 State–trait anxiety inventory (STAI)

The STAI contains two subscales, state anxiety inventory (STAI-S) and trait anxiety inventory (STAI-T), with a total of 40 items (21). The scale is rated on a 4-point Likert scale (1 = almost never to 4 = almost always). The total scores were calculated for each subscale. Higher scores indicate higher levels of anxiety. STAI-T scores were used for group allocation, while STAI-S scores were used to evaluate the immediate state anxiety following control and exercise sessions. The Chinese version of STAI was used in the present study, with the Cronbach’s *α* = 0.82 (STAI-S) and 0.83 (STAI-T).

### 2.3 Cardiorespiratory fitness assessment

Estimated cardiorespiratory fitness was assessed on a bicycle ergometer (ergoselect 200, ergoline, Germany) using the YMCA cycle ergometry protocol, with maximal oxygen consumption (VO_2peak_) estimated. The pedal speed was maintained at 50 rpm. The entire testing protocol consisted of two to three 3-min stages. During the first stage, participants were required to cycle at a load of 25 W. Workload in subsequent stages was determined by the heart rate at the end of each stage. The protocol was terminated until the steady state heart rate raised to 110 and 150 bpm. Maximal heart rate was estimated using the formula (220-age). VO_2peak_ was calculated using an established formula (24).

### 2.4 Stroop task

The color-word Stroop task was administrated using E-prime 2.0. Chinese word meaning and ink color stimuli were red, green, blue and yellow. Those combined stimuli consisted of two conditions: congruent and incongruent. In the congruent condition, the Chinese word meaning and ink color were presented in the same color [e.g., “红(red)” presented in the red ink color]. In the incongruent condition, the words were presented in one of the other three colors [e.g., “红(red)” presented in the blue ink color]. Participants were required to respond as quickly and accurately as possible to color naming and ignore the word meaning by pressing one of four response keys (D, F, J, K) on the keyboard. Stimuli were presented at the center of a 17-inch computer screen. Each trial began with a white fixation cross for 1 s, followed by a color-word stimulus after a variable interstimulus interval of 1–1.5 s. The stimulus remained on the screen for a maximum of 1.5 s or until a response was made. Congruent and incongruent trials were presented with equal probability (50%) in pseudorandomized order. The task consisted of six blocks of 72 trials. Each block was followed by a short break. 12 trials of each block were excluded because of the priming effect. Response times for correct trials and error rates were analyzed separately for congruent and incongruent conditions.

### 2.5 ERP recording and processing

Continuous electroencephalogram (EEG) was recorded during the Stroop task from 64 scalp sites using an ANT Neuro eego device (ANT B. V., Enschede, the Netherlands) with the international 10–20 system. Electrode impedances were keeped below 10 kΩ. During recording, the EEG was referenced online to the CPz and digitized at a sampling rate of 500 Hz. EEG data were offline performed in MATLAB using EEGLAB v2021.0 and ERPLAB 8.0.0. The data were re-referenced to the average of the left and right mastoids.

The EEG data were filtered offline with a band-pass of 0.1–30 Hz. Independent component analysis (ICA) was performed to decompose EEG signals and identify ocular artifact components associated with eye movements and blinks. Components related to ocular artifacts were identified based on visual inspection of component activations and scalp topographies and were subsequently removed. Stimulus-locked ERP epochs were extracted from −200 to 800 ms and baseline corrected over a window of −200 to 0 ms prior to the stimulus onset. Trials with incorrect responses or artifacts exceeding ± 75 μV at any electrode were excluded before averaging. The percentages of rejected trials after artifact detection were 13.82 and 32.67% (in the control and exercise sessions, respectively) for the LTA group, and 10.03 and 18.08% (in the control and exercise sessions, respectively) for the HTA group. Each Stroop condition originally contained 180 trials, and approximately 121–162 trials were retained for ERP averaging in each condition after artifact rejection. Mean amplitudes of the N400 (350–450 ms) and SP (600–800 ms) components were calculated separately within their respective time windows. Based on previous Stroop ERP studies showing a fronto-central distribution for conflict-related N400 activity and a more posterior distribution for the SP (15, 18), N400 amplitudes were analyzed at Fz and Cz, whereas SP amplitudes were analyzed at Cz and Pz.

### 2.6 Experimental procedures

First, participants were instructed and completed questionnaires to assess demographic information and trait anxiety. Eligible participants were required to sit quietly and equipped with a heart rate monitor (polar V800, Polar Electro Oy, Finland) to measure resting heart rate. The participants practiced several blocks of 24 Stroop trials until they achieved an accuracy rate of at least 90%. Estimated cardiorespiratory fitness was then assessed using the YMCA cycle ergometry protocol.

Second, all participants completed both exercise and control sessions in a counterbalanced order. 11 participants in the HTA group and 10 participants in the LTA group completed the control session first, whereas the remaining participants completed the exercise session first. The interval between sessions was 2–3 days (mean ± SD: 2.45 ± 0.50 days). The exercise session lasted 30-min and included a 5-min warm-up, a 20-min steady-state at 60–70% of maximal heart rate, and a 5-min cool-down. The workload started at 25 W and was adjusted until heart rate reached 60–70% of maximal heart rate. Pedal cadence was maintained at 70 rpm. Heart rate and ratings of perceived exertion (RPE; 6 = not exertion at all to 20 = extremely hard exertion) were measured every 3-min to ensure the moderate intensity exercise. During the control session, Participants seated rest for 30-min. Following each session, participants completed the STAI-S questionnaire, then performed the Stroop task while EEG signals were recorded simultaneously. After completion all sessions, all participants were asked and briefed on the purpose of study.

### 2.7 Statistical analysis

All analyses were conducted using SPSS 20.0. An independent sample *t* test was conducted to examine the effectiveness of group allocation. To evaluate the exercise manipulation, heart rates were analyzed using a 2 × 2 × 2 mixed analysis of variance (ANOVA) with Group (HTA, LTA) as a between-subject factor, and Treatment (exercise, control) and Measurement time (resting heart rate, exercise heart rate) as within-subject factors. Response times and error rates in the Stroop task were analyzed using a 2 × 2 × 2 mixed ANOVA with Group (HTA, LTA) as a between-subject factor, and Treatment (exercise, control) and Stroop task condition (congruent, incongruent) as within-subject factors. Conflict effects were calculated separately for response time and error rate as the difference between incongruent and congruent trials [e.g., (mean error rate in incongruent trials)-(mean error rate in congruent trials)] in the control and exercise sessions. The conflict scores were analyzed using a 2 × 2 mixed ANOVA with Group (HTA, LTA) as a between-subject factor and Treatment (exercise, control) as within-subject factors.

For the EEG recordings, the mean amplitudes for each component (N400 and SP) were analyzed using a 2 × 2 × 2 mixed ANOVA with Group (HTA, LTA) as a between-subject factor, and Treatment (exercise, control) and Stroop task condition (congruent, incongruent) as within-subject factors. Difference amplitudes between incongruent and congruent conditions [e.g., (mean amplitude in incongruent condition)-(mean amplitude in congruent condition)] were calculated for the N400 and SP and analyzed using a 2 × 2 mixed ANOVA with Group (HTA, LTA) as a between-subject factor and Treatment (exercise, control) as within-subject factors. *η*_p_^2^ is reported as an effect size measure. When the assumption of sphericity was violated, degrees of freedom and *p* values were corrected using the Greenhouse–Geisser correction. Multiple comparisons were adjusted by using Bonferroni correction. An alpha level of statistical significance was set as 0.05.

## 3 Results

### 3.1 Sample characteristics

There was a significant difference in the STAI-T scores between the LTA (29.76 ± 4.37) and HTA (54.24 ± 5.30) groups [*t*_(40)_ = 16.35, *p* < 0.001], indicating the effectiveness of grofup allocation. There was no significant difference in the VO_2peak_ between the LTA and HTA groups [*t*_(40)_ = 0.28, *p* = 0.78], indicating no difference in the cardiorespiratory fitness between groups.

### 3.2 Exercise treatment manipulation

To investigate the effectiveness of the exercise manipulation, heart rates were assessed before and during the exercise and control sessions in both groups. The three-way ANOVA revealed significant main effects of Treatment [*F*_(1, 40)_ = 3512.40, *p* < 0.001, *η*_p_^2^ = 0.99] and Measurement time [*F*_(1, 40)_ = 4739.78, *p* < 0.001, *η*_p_^2^ = 0.99], as well as a significant interaction between Treatment and Measurement time [*F*_(1, 40)_ = 4417.42, *p* < 0.001, *η*_p_^2^ = 0.99]. The main effect of Group was not significant [*F*_(1, 40)_ = 3.69, *p* = 0.09]. Bonferroni-adjusted pairwise comparisons showed that heart rate during the exercise session (*M* = 136.64, SEM = 0.86 bpm) was significantly higher than resting heart rate (*M* = 65.64, SEM = 0.57 bpm), whereas no significant difference was observed between resting heart rate (*M* = 65.52, SEM = 0.80 bpm) and heart rate during the control session (*M* = 65.21, SEM = 0.76 bpm). These results indicate that the exercise manipulation successfully elevated heart rate in both the LTA and HTA groups. In addition, the average rating of RPE (HTA: *M* = 13.43, SEM = 0.68; LTA: *M* = 13.14, SEM = 0.57) and exercise heart rate (HTA: 68.76% of the maximal heart rate; LTA: 67.94% of the maximal heart rate) confirmed that the exercise session was performed at moderate intensity.

### 3.3 State anxiety score

The two-way ANOVA revealed significant main effects of Treatment [*F*_(1, 40)_ = 40.68, *p* < 0.001, *η*_p_^2^ = 0.50] with lower state anxiety scores after the exercise session (*M* = 28.67, SEM = 0.72) than after the control session (*M* = 33.10, SEM = 0.90), and Group [*F*_(1, 40)_ = 62.01, *p* < 0.001, *η*_p_^2^ = 0.61] with higher state anxiety scores in the HTA group (*M* = 36.69, SEM = 1.04) than in the LTA group (*M* = 25.07, SEM = 1.04). A significant interaction between Treatment and Group [*F*_(1, 40)_ = 15.82, *p* < 0.001, *η*_p_^2^ = 0.28] was also observed. Bonferroni-adjusted pairwise comparisons showed state anxiety scores were higher in the HTA group than in the LTA group following both the exercise session (HTA: *M* = 33.10, SEM = 1.12, LTA: *M* = 24.24, SEM = 0.91, *p* < 0.001) and the control session (HTA: *M* = 40.29, SEM = 1.58, LTA: *M* = 25.90, SEM = 0.87, *p* < 0.001). State anxiety scores were significantly lower after exercise than after the control session in the HTA group (*p* < 0.001), whereas this difference was not significant in the LTA group (*p* = 0.10).

### 3.4 Behavioral task performance

#### 3.4.1 Response time

Response times in the Stroop task were analyzed after the control and exercise sessions in the LTA and HTA groups (Table 1). The three-way ANOVA revealed a significant main effect of Treatment [*F*_(1, 40)_ = 106.54, *p* < 0.001, *η*_p_^2^ = 0.73] with longer response times observed in the control session (*M* = 747.03, SEM = 10.00) compared with the exercise session (*M* = 663.57, SEM = 11.55). A significant main effect of Stroop task condition was also observed [*F*_(1, 40)_ = 495.73, *p* < 0.001, *η*_p_^2^ = 0.93], reflecting the typical Stroop effect, with longer response times in the incongruent condition (*M* = 755.91, SEM = 10.43) than in the congruent condition (*M* = 654.68, SEM = 10.12). The main effect of Group and all interaction effects were not significant (see Table 2).

**Table 1:** Stroop task performance under congruent and incongruent conditions across treatment sessions and anxiety groups (*M ± SEM*).

Measure	LTA group				HTA group			
	Control session		Exercise session		Control session		Exercise session	
	Cong	Incong	Cong	Incong	Cong	Incong	Cong	Incong
Response time (ms)	696.33 (17.28)	800.24 (16.73)	624.57 (18.22)	725.43 (19.63)	693.55 (11.45)	797.99 (12.23)	604.27 (13.09)	699.99 (15.33)
Error rate (%)	4.46 (0.68)	6.73 (0.65)	2.43 (0.40)	4.37 (0.63)	5.30 (0.97)	8.44 (1.09)	2.89 (0.52)	4.29 (0.68)

**Table 2:** Statistical results of behavioral results.

Measure	Response time			Error rate		
	*f*	*p*	*η*_p_^2^	*f*	*p*	*η*_p_^2^
Treatment	106.54	<0.001	0.73	23.06	<0.001	0.37
Stroop task condition	495.73	<0.001	0.93	62.91	<0.001	0.61
Group	0.40	0.53	0.01	0.87	0.36	0.02
Treatment × Stroop task condition	1.10	0.30	0.03	4.31	0.04	0.10
Treatment × Group	1.58	0.22	0.04	0.90	0.35	0.02
Stroop task condition × Group	0.06	0.80	0.002	0.10	0.76	0.002
Treatment × Stroop task condition × Group	0.26	0.62	0.006	2.00	0.17	0.05

The difference scores for response time between incongruent and congruent conditions were calculated and analyzed in both the LTA and HTA groups. The two-way ANOVA showed no significant main effect of Treatment [*F*_(1, 40)_ = 1.10, *p* = 0.30] and Group effect [*F*_(1, 40)_ = 0.06, *p* = 0.80], and no interaction effect [*F*_(1, 40)_ = 0.26, *p* = 0.62].

#### 3.4.2 Error rate

Error rates in the Stroop task were analyzed after the control and exercise sessions in the LTA and HTA groups (Table 1). The three-way ANOVA revealed a significant main effect of Treatment [*F*_(1, 40)_ = 23.06, *p* < 0.001, *η*_p_^2^ = 0.37] with higher error rates in the control session (*M* = 6.23, SEM = 0.58) than in the exercise session (*M* = 3.49, SEM = 0.37). A significant main effect of Stroop task condition was also observed [*F*_(1, 40)_ = 62.91, *p* < 0.001, *η*_p_^2^ = 0.61] with higher error rates in the incongruent condition (*M* = 5.96, SEM = 0.46) than in the congruent condition (*M* = 3.77, SEM = 0.37). The Treatment × Stroop task condition interaction was significant [*F*_(1, 40)_ = 4.31, *p* = 0.04, *η*_p_^2^ = 0.10] (see Table 2). Bonferroni-adjusted pairwise comparisons showed that error rates were significantly lower after exercise than after the control session in both the congruent (exercise: *M* = 2.66, SEM = 0.33; control: *M* = 4.88, SEM = 0.59) and incongruent conditions (exercise: *M* = 4.33, SEM = 0.46; control: *M* = 7.58, SEM = 0.63). These findings indicate that acute moderate-intensity aerobic exercise generally facilitated Stroop task performance.

For the difference scores of error rate (incongruent minus congruent), the two-way ANOVA showed a significant main effect of Treatment [*F*_(1, 40)_ = 4.31, *p* = 0.04, *η*_p_^2^ = 0.10] with higher difference scores in the control session (*M* = 2.70, SEM = 0.42) than in the exercise session (*M* = 1.67, SEM = 0.31). Neither the main effect of Group [*F*_(1, 40)_ = 0.09, *p* = 0.76], nor the interaction effect [*F*_(1, 40)_ = 2.00, *p* = 0.17] was significant.

### 3.5 ERP

#### 3.5.1 N400

**Figure 1:**
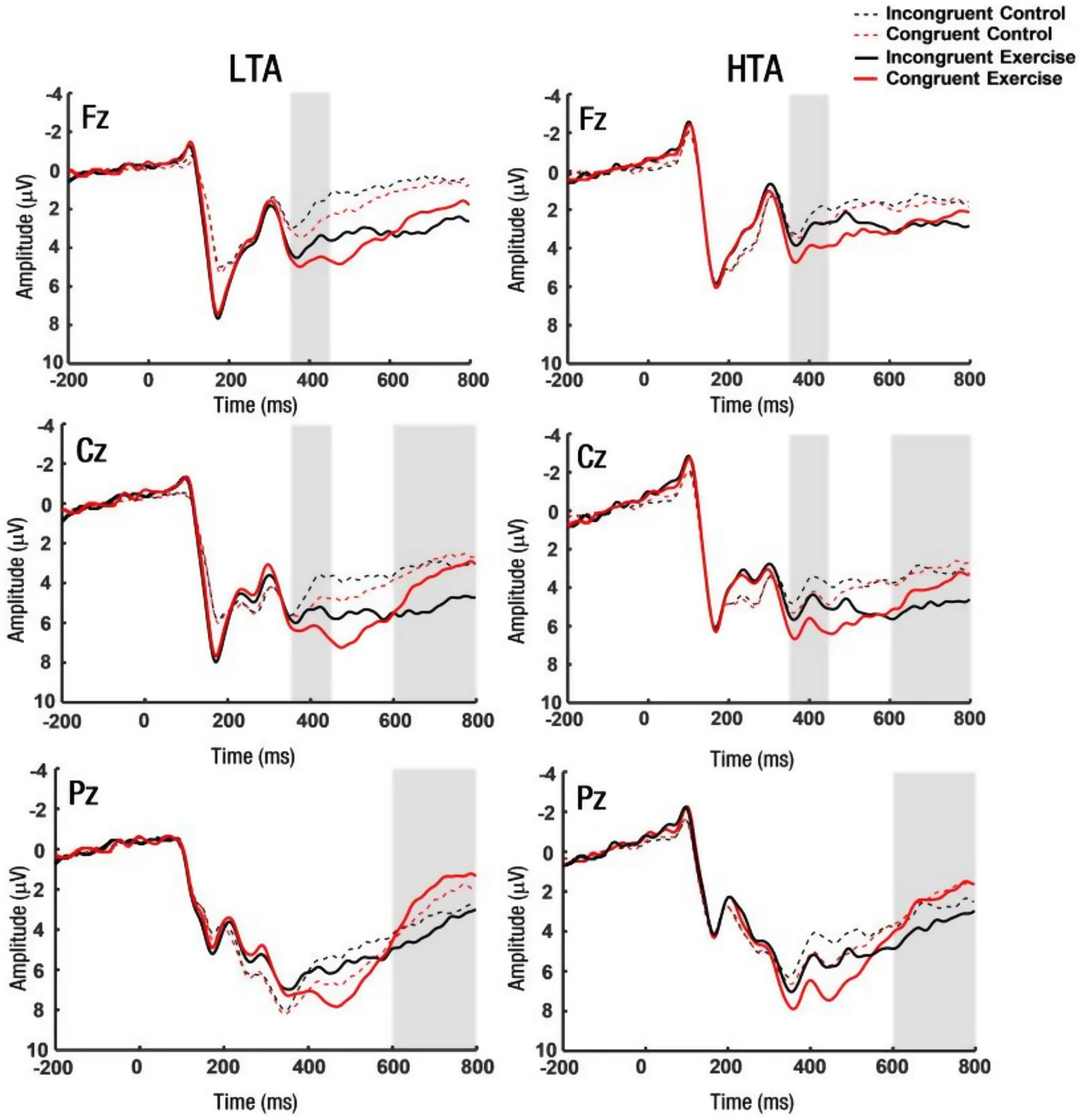
Grand-averaged ERP waveforms at Fz, Cz, and Pz by treatment (exercise/control) and Stroop task conditions (congruent/incongruent) in the LTA (left) and HTA (right) groups. Shaded gray areas indicate the predefined analysis windows for the N400 (350–450 ms, analyzed at Fz and Cz) and SP (600–800 ms, analyzed at Cz and Pz) components. LTA, Low trait anxiety; HTA, High trait anxiety.

**Figure 2:**
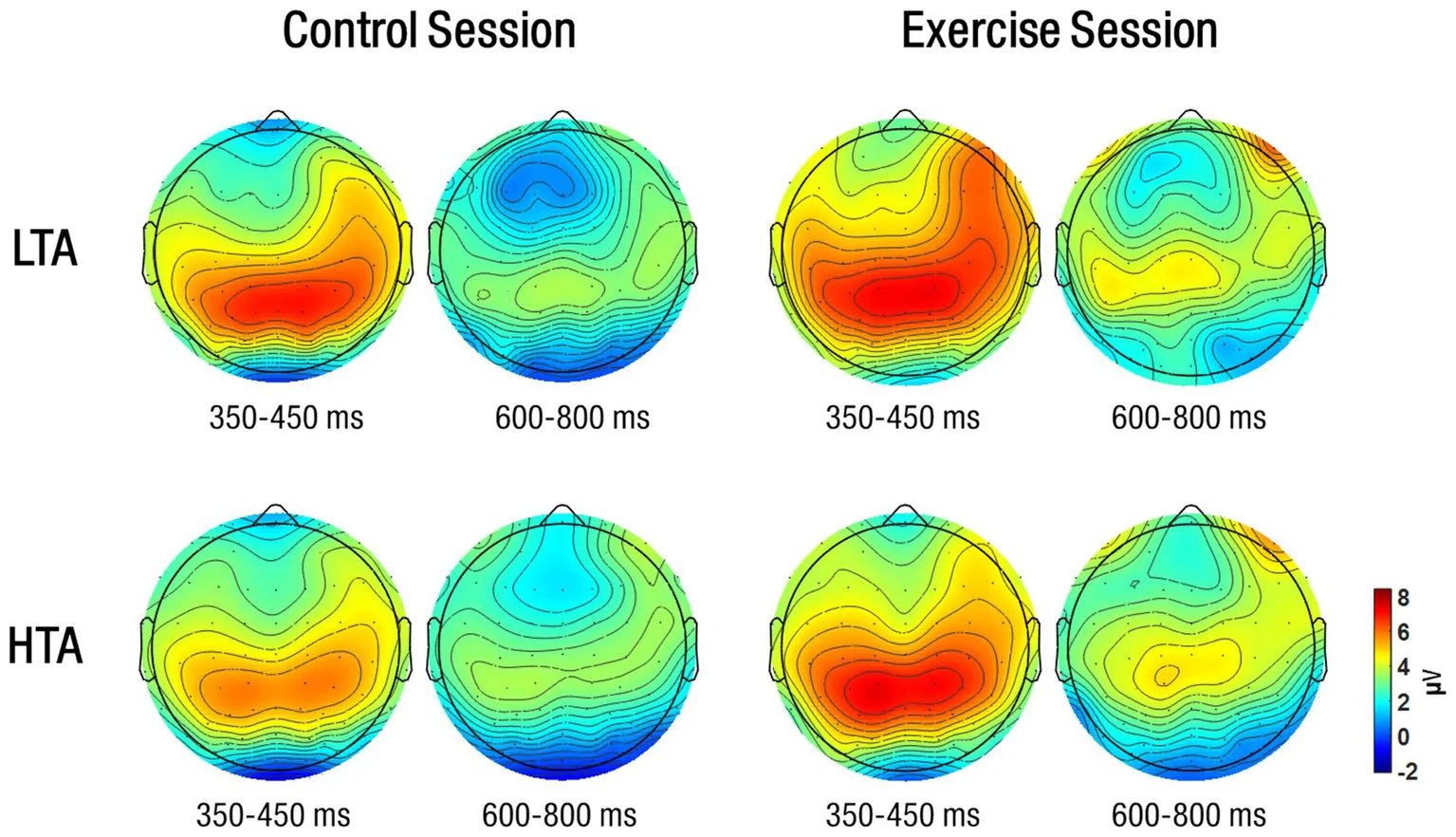
Topographic maps of N400 (350–450 ms) and SP (600–800 ms) amplitudes by treatment (exercise and control) in the LTA and HTA groups. LTA, low trait anxiety; HTA, high trait anxiety.

At Fz, the three-way ANOVA for mean N400 amplitude (Figures 1 and 2) revealed a significant main effect of Treatment [*F*_(1, 40)_ = 8.60, *p* = 0.006, *η*_p_^2^ = 0.18] with more negative mean N400 amplitudes after the control session (*M* = 2.63, SEM = 0.52) than after the exercise session (*M* = 3.85, SEM = 0.64). A significant main effect of Stroop task condition [*F*_(1, 40)_ = 29.40, *p* < 0.001, *η*_p_^2^ = 0.36] was also observed with a more negative mean N400 amplitudes in the incongruent condition (*M* = 2.83, SEM = 0.55) than in the congruent condition (*M* = 3.65, SEM = 0.55). No other significant main effects or interactions were observed.

At Cz, the three-way ANOVA for mean N400 amplitude revealed a significant main effect of Treatment [*F*_(1, 40)_ = 7.20, *p* = 0.01, *η*_p_^2^ = 0.15] with more negative mean N400 amplitudes after the control session (*M* = 4.50, SEM = 0.65) than after the exercise session (*M* = 5.48, SEM = 0.68). A significant main effect of Stroop task condition [*F*_(1, 40)_ = 27.97, *p* < 0.001, *η*_p_^2^ = 0.41] was also observed with a more negative mean N400 amplitudes in the incongruent condition (*M* = 4.51, SEM = 0.64) than in the congruent condition (*M* = 5.47, SEM = 0.65). No other significant main effects or interactions were observed (see Table 3).

**Table 3:** Statistical results of ERP results.

Measure	N400						SP					
	Fz			Cz			Cz			Pz		
	*f*	*p*	*η*_p_^2^	*f*	*p*	*η*_p_^2^	*f*	*p*	*η*_p_^2^	*f*	*p*	*η*_p_^2^
Treatment	8.60	0.006	0.18	7.20	0.01	0.15	5.98	0.019	0.13	0.07	0.79	0.002
Stroop task condition	23.35	<0.001	0.39	27.97	<0.001	0.41	4.15	0.048	0.09	15.73	<0.001	0.28
Group	0.06	0.81	0.001	0.17	0.68	0.004	0.17	0.69	0.004	0.22	0.64	0.006
Treatment × Stroop task condition	0.64	0.43	0.02	0.90	0.35	0.02	7.03	0.02	0.15	10.17	0.003	0.20
Treatment × Group	2.14	0.15	0.05	<0.001	0.998	<0.001	0.05	0.83	0.001	0.14	0.71	0.004
Stroop task condition × Group	0.001	0.98	<0.001	1.38	0.25	0.03	0.97	0.33	0.02	0.80	0.38	0.02
Treatment × Stroop task condition × Group	1.03	0.32	0.03	0.28	0.60	0.007	0.49	0.49	0.01	0.28	0.60	0.007

#### 3.5.2 SP

At Cz, the three-way ANOVA for mean SP amplitude revealed a significant main effect of Treatment [*F*_(1, 40)_ = 5.98, *p* = 0.02, *η*_p_^2^ = 0.13] with larger mean SP amplitude after the exercise session (*M* = 4.17, SEM = 0.68) than after control session (*M* = 2.96, SEM = 0.50). A significant main effect of Stroop task condition [*F*_(1, 40)_ = 4.15, *p* = 0.048, *η*_p_^2^ = 0.09] was also observed with larger mean SP amplitude in the incongruent condition (*M* = 3.78, SEM = 0.58) than in the congruent condition (*M* = 3.35, SEM = 0.53). A significant interaction effect between Treatment and Stroop task condition was observed [*F*_(1, 40)_ = 7.03, *p* = 0.01, *η*_p_^2^ = 0.15]. Bonferroni-adjusted pairwise comparisons showed that the incongruent condition elicited larger SP amplitudes than the congruent condition after the exercise (incongruent: *M* = 4.64, SEM = 0.71; congruent: *M* = 3.70, SEM = 0.68, *p* = 0.005), but not after the control session (incongruent: *M* = 2.92, SEM = 0.53; congruent: *M* = 3.01, SEM = 0.51, *p* = 0.72). In addition, SP amplitudes were larger after the exercise than after the control session in the incongruent condition (*p* = 0.001), but not in the congruent condition (*p* = 0.23). No other significant main effects or interactions were observed (see Table 3). For the SP difference amplitudes (incongruent minus congruent), the two-way ANOVA showed a significant main effect of Treatment at Cz [*F*_(1, 40)_ = 8.36, *p* = 0.006, *η*_p_^2^ = 0.17] with larger difference amplitudes after the exercise session (*M* = 0.94, SEM = 0.32) than after the control session (*M* = −0.09, SEM = 0.25). Neither the main effect of Group [*F*_(1, 40)_ = 0.86, *p* = 0.36], nor the interaction effect [*F*_(1, 40)_ = 0.58, *p* = 0.45] was significant.

At Pz, the three-way ANOVA for mean SP amplitude revealed a significant main effect of Stroop task condition [*F*_(1, 40)_ = 15.73, *p* < 0.001, *η*_p_^2^ = 0.28] with larger mean SP amplitude in the incongruent condition (*M* = 3.21, SEM = 0.50) than in the congruent condition (*M* = 2.40, SEM = 0.44). There was a significant interaction effect between Treatment and Stroop task condition [*F*_(1, 40)_ = 10.17, *p* = 0.003, *η*_p_^2^ = 0.20]. Bonferroni-adjusted pairwise comparisons showed that the incongruent condition elicited larger SP amplitudes than the congruent condition after exercise (incongruent: *M* = 3.43, SEM = 0.62; congruent: *M* = 2.08, SEM = 0.55, *p* < 0.001), but not after the control session (incongruent: *M* = 2.99, SEM = 0.46; congruent: *M* = 2.73, SEM = 0.44, *p* = 0.29).

Pearson correlation analysis was conducted to examine the association between behavioral conflict scores (error rate difference scores) and SP difference amplitudes at Cz. During the exercise session, behavioral conflict scores were positively correlated with SP difference amplitudes (*r* = 0.33, *p* = 0.04).

## 4 Discussion

This study examined the acute effects of a single 30-min bout of moderate-intensity aerobic exercise session (60–70% of maximal heart rate) on state anxiety and inhibitory control in individuals with different levels of trait anxiety, using behavioral measures and stimulus-locked ERP components. The results showed that acute moderate-intensity aerobic exercise reduced state anxiety in the HTA group but not in the LTA group. Notably, the HTA group exhibited higher state anxiety than the LTA group following both the control and exercise sessions. Acute moderate-intensity aerobic exercise also facilitated Stroop task performance, as reflected by faster response times and lower error rates across task conditions. At the neural level, acute aerobic exercise was associated with less negative N400 amplitudes and larger sustained positive potential (SP) amplitudes during the Stroop task in both groups. Consistent with previous studies, reductions in the state anxiety following acute moderate-intensity aerobic exercise tend to be greater in individuals with a higher initial anxiety levels (19, 25). The absence of a significant reduction in the LTA group may be related to their relatively low baseline state anxiety levels, leaving less room for further reduction. However, alternative explanations, such as differences in emotion regulation capacity or physiological stress responsivity, cannot be excluded because relevant physiological measures were not assessed in the present study.

Previous studies have found that acute moderate-intensity aerobic exercise exerts an immediate and broadly facilitative effect on cognitive performance in young adults (7, 13). More broadly, previous studies suggest that 20–40 min of moderate-intensity aerobic exercise can improve executive function, particularly inhibitory control assessed using Stroop and Flanker tasks (8, 26), with benefits reported in healthy populations (5, 27), as well as in individuals with clinical disorders (6, 28). The present study extends this evidence by showing that young adults benefited from acute exercise across trait-anxiety levels. This finding is consistent with previous research in college-aged adults showing that acute moderate-intensity aerobic exercise improved inhibitory control in both high- and low-anxious individuals (14). Together, these findings suggest that acute exercise-related cognitive benefits may generalize across anxiety levels during young adulthood. However, this pattern may differ across developmental stages. A previous study reported that trait anxiety moderated cognitive responses to acute exercise in children (29), suggesting that the relationship between anxiety and exercise-induced cognitive benefits may vary across developmental stages. One possible explanation is that executive-control systems, including inhibitory control and cognitive flexibility, continue to mature throughout childhood and adolescence, which may alter the extent to which acute exercise influences cognitive performance (30, 31). Future studies directly comparing children, adolescents, and young adults are needed to determine whether developmental stage moderates the interaction between trait anxiety and the cognitive effects of acute exercise.

ERP findings provide further insight into the temporal neural processes associated with behavioral changes following acute aerobic exercise. Previous ERP studies have identified the N400 component as being sensitive to congruency manipulation and conflict-related processing during the Stroop task (15, 18). In the present study, the incongruent condition elicited more negative mean N400 amplitudes than the congruent condition, consistent with previous findings showing enhanced N400 responses under conditions involving greater stimulus–response conflict (15). This pattern further supports the sensitivity of the N400 to conflict-related processing in the present Stroop paradigm. Acute moderate-intensity aerobic exercise also modulated mean N400 amplitude, with less negative N400 amplitudes observed following exercise than during the control session. This neural modulation accompanied faster response times and lower error rates following exercise, suggesting that acute aerobic exercise may influence the neural processes associated with conflict-related cognitive control. However, given that ERP amplitude changes do not directly establish causal mechanisms, the altered N400 response should be interpreted as an electrophysiological correlate of modified conflict-related processing rather than direct evidence that exercise enhances the efficiency of conflict monitoring.

The results also showed that acute aerobic exercise elicited larger SP amplitudes relative to the control session, particularly for incongruent trials. Because the SP has been associated with later stages of conflict-related processing and response adjustment (32), these findings suggest that acute aerobic exercise may be associated with modulation of later-stage cognitive control processes under conditions of higher task demand. One possible explanation is that congruent trials involve relatively low levels of conflict and therefore provide less opportunity for exercise-related modulation to emerge. The absence of significant Group or Treatment × Group effect on SP amplitude suggests that this exercise-related neural modulation did not differ as a function of trait-anxiety level in the present task. However, the current Stroop paradigm involved non-emotional stimuli and may therefore have been relatively insensitive to anxiety-related differences in the processing of emotionally salient information. Prior studies have shown that HTA is associated with impaired inhibitory control, greater susceptibility to task-irrelevant information, and attentional bias toward emotionally salient stimuli (18, 33, 34). Future studies should therefore examine whether acute exercise produces differential cognitive and neural effects across trait-anxiety levels when inhibitory-control tasks incorporate emotionally relevant or threat-related stimuli.

Several limitations should be acknowledged. First, participants were recruited from a single university specializing in physical education, which may limit the generalizability of the findings to young adults with different educational backgrounds, habitual physical activity levels, or clinical anxiety populations. Second, the present study examined only the acute effects of a single 30-min bout of moderate-intensity cycling exercise; therefore, whether repeated exercise training or other exercise modalities, intensities, and durations produce similar affective and cognitive benefits remains unclear. Third, although ERP measures provided insight into the temporal dynamics of cognitive processing, the present findings cannot establish direct causal relationships between exercise-related ERP changes and improvements in inhibitory control. Future studies should employ longitudinal randomized controlled designs, systematically compare different exercise modalities, intensities, and durations, incorporate physiological measures such as cortisol and heart-rate variability, and include participants across different developmental stages. Such approaches may help clarify the factors contributing to individual differences in affective, behavioral, and neural responses to acute exercise.

In conclusion, a single bout of moderate-intensity aerobic exercise selectively reduced state anxiety in young adults with HTA and broadly improved inhibitory control performance in young adults with both LTA and HTA. These behavioral benefits were accompanied by exercise-related alterations in ERP indices associated with different stages of conflict processing. Acute moderate-intensity aerobic exercise may therefore be a practical strategy for transiently supporting inhibitory control in healthy young adults across different levels of trait anxiety.

## Data Availability

The raw data supporting the conclusions of this article will be made available by the authors, without undue reservation.
